# Royal Medical and Chirurgical Society

**Published:** 1897-10-30

**Authors:** 


### ROYAL MEDICAL AND CHIRURGICAL SOCIETY.

### Perforation of the Aorta by Calcareous Tracheal Glands.

Dr. Sydney Phillips described a remarkable case in which frequently repeated severe hæmoptysis occurring for fifteen months before death, was found on post-mortem examination to have been caused by erosion and perforation of the under-side of the commencement of the arch of the aorta by a group of rough calcareous lymphatic glands attached to the trachea about its bifurcation. The course of events in this very curious case appeared to have been as follows: (*a*) Calcareous metamorphosis and matting together of a group of lymphatic glands, forming a rigid tumour attached to the tracheal bifurcation; (*b*) Acute inflammation and suppuration in the connective tissue of this tumour, forming an encysted abscess; (*c*) perforation of the right bronchus by pus from this abscess, leaving a permanent bronchial fistula; (*d*) subsequent erosion and perforation of the wall of the arch of the aorta by pressure of the calcareous glands; (*e*) recurrent hæmoptysis from the aorta into the empty abscess cavity, and thence through the bronchial fistula into the trachea; (*f*) death from hemiplegia, the result probably of thrombosis from the effect of the repeated hæmorrhages. Three and a half years before the fatal termination of the case there had been a very severe illness, with intense pain on the right side of the chest, fever, sweats, and rigors, an incessant violent cough, loud systolic murmurs over the base of the heart, and ultimately the rapid development of extreme effusion of clear fluid in the left chest. After this had been aspirated eleven times it was opened and drained by Mr. Godlee, and after this the patient got about in moderate but never perfect health for 18 months, when the hæmoptysis began. For 15 months this continued. He certainly lost on an average 16 ounces of blood a week, and, in all, the quantity lost must have been nine or ten gallons. But he went about till shortly before his death. A week before his death, after two days of very free bleeding, he became hemiplegic on the left side, unconsciousness supervened, and he died in this condition. So that even in the end, notwithstanding the hole in his aorta, he did not die directly from hæmorrhage.

### Pernicious Malarial Fevers at Sierra Leone.

Dr. George Thin showed a large number of specimens illustrating the appearances found on examining the tissues in four cases of fatal pernicious malarial fever which had occurred at Sierra Leone. The main points of interest were the facts that the parasites were principally found in the brain, and that in all the cases one stage of development of the parasite usually predominated, viz., the early stage. In regard to the apparent selection of the cerebral capillaries by the parasite, two points had to be considered—first, the small calibre of these vessels, and, second, the fact that the affected corpuscles appear to adhere to walls of the vessels, which does not seem to be the case in tertian and quartan fevers, the result, perhaps, of an increased toxic quality of the parasite in the pernicious fevers.

